# Calendrier de Sante, 1890

**Published:** 1890-01-18

**Authors:** 


### CALENDRIER DE SANTE 1890.

### SUPPLEMENT AU *JOURNAL D'HYGIENE.*

For a practical joke in the way of supplements commend us to the almanack for 1890 published by the *Journal d'Hygiène*. The contents of this document are so valuable that they cannot be taken all at once, but are divided between two sheets of inconvenient size, "one to be taken" from January to June, the other from July to December. They form a collection of quite disconnected medico-moral platitudes, baldly stated and illustrated. Presumably the *Journal d'Hygiene* finds its way into the poorest quarters, for we find here such advice as "Do not spare soap and warm water," "Always keep your skin clean," "Dirty clothes are as bad as a dirty skin," and so on, recommendations which we imagine are not directed at the medical profession or educated classes across the Channel. The principal illustration represents an aged man in a badly-made sack much too small for him, gazing at some chemical appliances, and modestly turning his back on the goddess "Higeia" who is teaching "l'art de bien se porter" to a naked urchin. In the background is a Greek temple and a pair of scales, apparently prophetic of railway stations and automatic weighing machines. "Change here for Olympus" would be an apt title for this picture, the time chosen being when the operation of changing is half over. Attention should also be drawn to a weird cow with eczema, emblematic of vaccination.

A "Pharmacie de Famille," and an adequate number of advertisements, the whole being printed in shades of green, complete this quaint specimen of French journalistic almanacks.

